# ECG-Gated CCTA in the Assessment of Post-Procedural Complications

**DOI:** 10.3390/diagnostics13152500

**Published:** 2023-07-27

**Authors:** Carlo Liguori, Giulia Lassandro, Giovanni Ferrandino, Stefano Giusto Picchi, Stefania Tamburrini, Gabriella Toro, Fabio Tamburro, Salvatore Masala, Mariano Scaglione

**Affiliations:** 1 Department of Radiology, Ospedale del Mare-ASL NA1 Centro, Via Enrico Russo 11, 80147 Naples, Italy; giulia.lassandro@gmail.com (G.L.); stefanopicchi@libero.it (S.G.P.); tamburrinistefania@gmail.com (S.T.);; 2 Department of Medical, Surgical and Experimental Sciences, University of Sassari, 07100 Sassari, Italy; samasala@uniss.it (S.M.);; 3 Department of Radiology, James Cook University Hospital, Middlesbrough TS4 3BW, UK

**Keywords:** cardiac CT, coronary artery disease, coronary computed tomography angiography, transcatheter aortic valve implantation, pacemaker, percutaneous coronary intervention

## Abstract

Introduction: The aim of our study was to assess the role of ECG-gated coronary CT angiography (CCTA) in the diagnosis, imaging follow-up, and treatment guidance in post-procedural/surgical interventions in the heart and thoracic aorta (PTCA, TAVI, PMK/ICD placement, CABGs). Materials and Methods: We retrospectively evaluated 294 ECG-gated CCTA studies performed in our center from January 2020 to January 2023. CCTA studies were acquired to detect/exclude possible complications related to the endovascular or surgical procedure. Results: There were 27 cases (9.2%) of post-procedural complications. Patients enrolled in the study were 18 males and 9 females (male/female ratio: 2), with age ranging from 47 to 86 years (mean age, 68.3 years). Among percutaneous coronary intervention (PCI) complications, coronary intimal dissection with ascending aorta involvement was found to be the most frequent complication after PTCA (22.2%). Vascular wall pseudoaneurysm formation (11.1%) and coronary stent misalignment or displacement (14.8%) were complications less frequently encountered after PTCA. Right atrial or ventricular perforation with associated hemopericardium were the most common complications (18.5%) after pacemaker implantation. Complications encountered after aortic valve interventions were loosening and dislocation of the prosthesis associated with aortic root pseudoaneurysm (7.4%), para-valvular leak (11.1%), and hemopericardium (7.4%). In one patient who underwent transcatheter repair of patent foramen ovale (3.7%), CTTA detected the dislocation of the Amplatzer septal occluder. Conclusions: ECG-gated CCTA is a fundamental diagnostic tool for the detection of post-procedural endovascular/surgical complications to enable optimal patient management. Radiologists must be familiar with the use of cardiac synchronization in the course of CT and must be aware of all possible complications that can occur in the context of acute settings or routine follow-up studies.

## 1. Introduction

Coronary artery and aortic intervention procedures are used in the treatment of a wide spectrum of diseases affecting the heart and systemic vessels.

Cardiac and noncardiac complications can occur at variable times after procedures, with the clinical presentation ranging from asymptomatic to devastating.

Some of the cardiac complications can be seen with any cardiac interventions and are related to a possible direct damage to the coronaries or cardiac structures, while others are specific to the interventional treatment. Some noncardiac complications must be taken into account and can be found on the vascular access site (pseudoaneurism formation or hematoma) or on a distant site because of material embolization (stroke, or parenchymal injury). These complications occur in variable time frames after the procedure.

Invasive coronary angiography represents the gold-standard technique for the diagnosis of coronary artery disease (CAD), but nowadays coronary CT angiography (CCTA) is considered the first-choice non-invasive alternative method, mainly for patients presenting with chronic chest pain and in the acute phase for those presenting with low to intermediate pre-test risk [1].

CT is nowadays largely employed in several medical and research applications, becoming an irreplaceable tool for diagnostics and therapeutic planning.

The technical development of cardiac CT scanners has moved in two different directions in recent years: some vendors have implemented effective volume coverage for a single rotation, increasing the width of detector rows up to 16 cm, allowing for the acquisition of all the cardiac volume in a single beat.

On the other hand, other manufacturers have introduced, on the same system, two radiation tubes designed to work in a simultaneous manner: this option guarantees a significant increase in effective temporal resolution but makes possible the so called “high pitch-flash” acquisition, a solution to acquire all the data for the heart and coronaries in a single beat as well.

Both solutions have enabled volumetric imaging of the whole heart without artifacts, with a high temporal resolution and optimal diagnostic performance. Technological implementation significantly reduced the image-acquisition time and, consequently, the radiation dose and the volume of contrast injected [2].

Further advances have also been achieved by improving spatial and temporal resolution, making CCTA increasingly used in the diagnostic work-up of patients with suspected CAD [3,4].

Currently, ECG-gated CCTA is the gold-standard pre-procedural imaging technique for structural heart surgery and for planning a transcatheter aortic valve implantation (TAVI) [5].

ECG-gated CCTA assists interventional cardiologists, providing detailed anatomical information for the adequate selection of catheters, guidewires, stent sizes, and the correct timeline for multi-step revascularization [6].

CCTA also has a prognostic value for procedural outcomes, especially in cases of chronic total coronary artery occlusion [7].

However, although ECG-gated CCTA is part of preoperative work-ups [8,9], there are still few studies in the literature and no recommendation for its use in the follow-up of patients who have undergone cardiac surgery.

Procedures such as percutaneous transluminal coronary angioplasty (PTCA) and TAVI or pacemaker (PMK) positioning can still lead to potentially life-threatening complications.

Post-operative cardiac complications are rare but possible events with reported incidence ranging from 0.02% to 1% [10,11,12,13]. However, other studies affirm that the true incidence of these complications is higher (4–5%) because the problems are often unrecognized and underestimated [14,15,16].

The aim of our study was, first, to report our experience of the role of ECG-gated CCTA in the assessment of postoperative complications in patients undergoing cardiovascular procedures as a non-invasive imaging technique of choice for the diagnosis, management, and follow-up in post-procedural complications, and second, to review the extensive international literature on the topic. The originality of this work is represented by the inclusion of a population of patients undergoing a wide variety of coronary and extracoronary endovascular procedures, all of whom are reevaluated with cardiosynchronized CT examinations. In fact, CT-gating has up to now been considered an indispensable aid for an adequate evaluation of the coronary artery and the ascending aortic wall, but its use is not yet extensively codified for the analysis of patients with extracoronary heart complications.

## 2. Materials and Methods

### 2.1. Patients

We retrospectively evaluated a total of 294 CCTA with ECG-gating (retrospectively modulated acquisition) studies performed in our center from January 2020 to January 2023 after interventional/endovascular procedures or cardiac surgery. ECG-gating CCTA was performed in emergency settings if patients showed symptoms consistent with an acute complication or in standard settings as the regular follow-up (30 days to 6 months) after the procedure.

Two radiologists independently reviewed the CCTA exams on a dedicated workstation, blinded to the identity and the anamnestic data of all the patients.

Informed consent was obtained from all patients at the time of the exam.

### 2.2. CT Imaging

CCTA exams were performed using two multi-detector CT systems (SOMATOM Drive 256-channels, Dual Source, Siemens, Forchheim, Germany and Aquilion Prime-160 channels, Canon Medical Systems, Otawara, Japan) with modulated retrospective ECG-gating and an iterative reconstruction algorithm for radiation and contrast-dose reduction.

Oral nitrates were always used to widen coronary lumen, and β-receptor blocker medication was employed in case of heart rate >65 bpm.

The CCTA protocol at our institution consists of a preliminary non-contrast, prospectively gated, low-radiation-dose CT chest scan to define the correct anatomical volume for the following CCTA and to evaluate collateral findings (lungs, pleura, mediastinum, chest wall, etc.). After bolus tracking in the ascending aorta (100 UH) and breathing command (6 s), a bolus ranging from 80 mL to 100 mL (according to the scanner characteristics and acquisition length) of a nonionic contrast agent (370–400 mgI/mL concentration) is injected at the rate of 4–5 mL/s, followed by a 40-mL saline bolus at the same injection rate used for contrast. The CCTA is acquired at the thinnest collimation (0.5–0.625 mm in cranio-caudal direction with retrospective ECG-gating in the arterial phase, using tube–current modulation (maximum mAs between 40% and 80% of the cardiac cycle). The contrast volume and velocity injection were adapted to the IDR (iodine delivery rate) target.

For cardiac structure evaluation, in all cases, a multiphase reconstruction of 40–80% of the cardiac cycle was made, with a 10% increment, including the best systolic and best diastolic phase.

All arterial ECG-gated acquisition was followed by a later non-gated scan to assess or exclude medium-contrast vessel extravasation. After data acquisition, the image-data set was sent to the picture communication system (PACS) and reported with dedicated imaging-reporting software (Syngovia workstation, Siemens Healthcare, Forchheim, Germany).

## 3. Results

Post-procedural complications were encountered in 27/294 patients (9.2%). The 27 patients enrolled in the study were 18 males and 9 females (male/female ratio: 2), with ages ranging from 47 to 86 years (mean age 68.3 years). Demographic data and complications correctly diagnosed through CTA and grouped according to clinical presentation are summarized in Table 1.

Among 27 patients presenting with post-procedural complications, 10 (37%) showed acute manifestations in an emergency setting, and 17 (63%) were asymptomatic; diagnosis was made during a routinely performed follow-up study.

**Figure 1:**
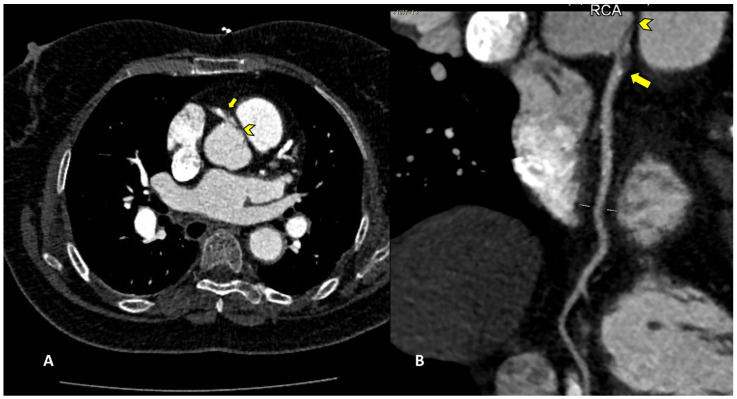
CCTA, curved planar reconstruction: (**A**) ascending aorta dissection involvement (arrowhead); (**B**) RCA ostial dissection can be noted (arrow).

Among percutaneous coronary intervention (PCI) complications, coronary intimal dissection was found to be the most frequent event after PTCA (6/27 patients, 22.2%), associated with involvement of the ascending aorta (Figure 1). All coronary dissections encountered were noted in an emergency setting, and all of them involved the right coronary ostia, with a constant flap extension to the valsalva sinus and ascending aorta wall (less than 40 mm distance from the ostium).

**Figure 2:**
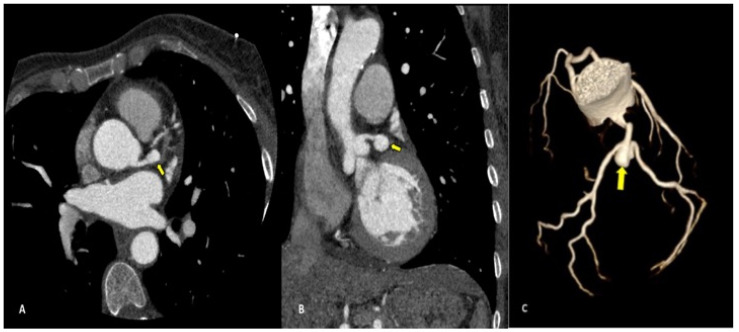
CCTA performed as follow-up acquisition after a coronary PTCA in an asymptomatic patient: axial (**A**) and coronal (**B**) multiplanar reconstructions showing distal left-main pseudoaneurysm (arrow) dimensions; 2.2 × 1.2 cm. Volume-rendering (**C**) image confirms the vascular sac at the edge of left main trunk.

**Figure 3:**
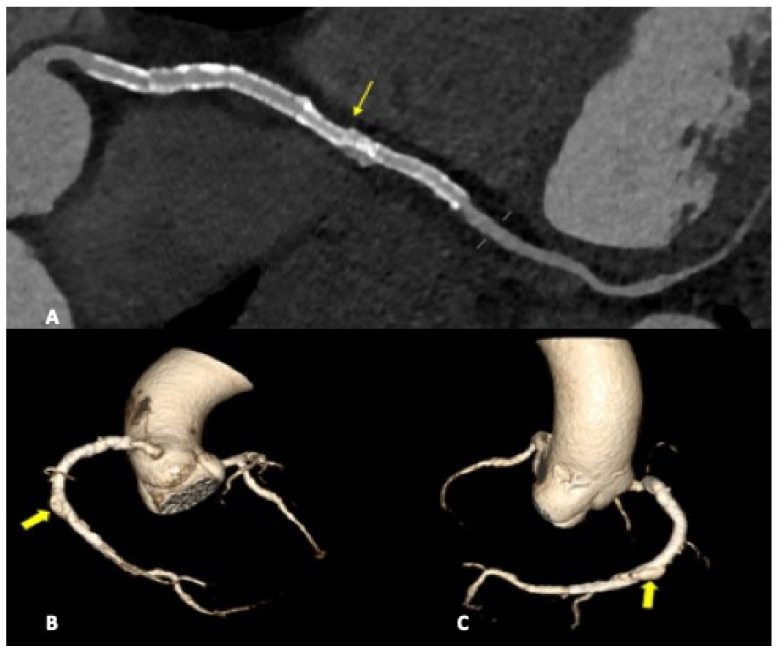
CCTA, a curved planar reconstruction of RCA (**A**) and volume-rendering reconstruction of coronary tree (**B**,**C**). A: multiple stents in RCA can be seen. The most distal stent is misaligned (light arrow), with vascular dilatation and endoleak in the same vascular tract. (**B**,**C**): medium-contrast extravasation outside the stent in the distal RCA determines a constrast sacciform collection outside the stent meshes as an endoleak type III formation (heavy arrows).

**Figure 4:**
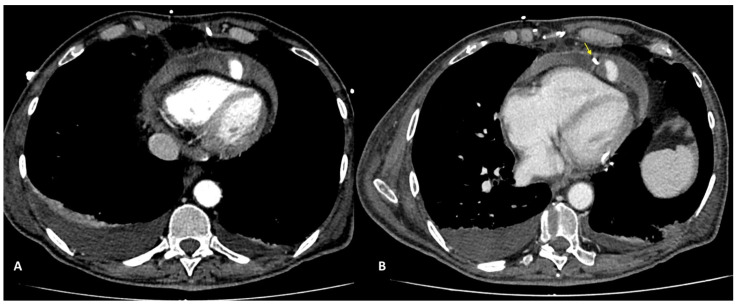
CCTA, axial planes, arterial-gated (**A**) and late non-gated (**B**) phases: medium-contrast blush can be noted in both arterial and venous phases with hemopericardium. In (**B**), the tip of the pericardial drainage catheter can also be noted (arrow).

Complications less frequently presented after PTCA were vascular-wall pseudoaneurysm formation (3/27 patients, 11.1%) (Figure 2) or coronary stent migration or displacement (4/27 patients, 14.8%), which were always diagnosed in non-symptomatic patients (Figure 3 and Figure 4).

**Figure 5:**
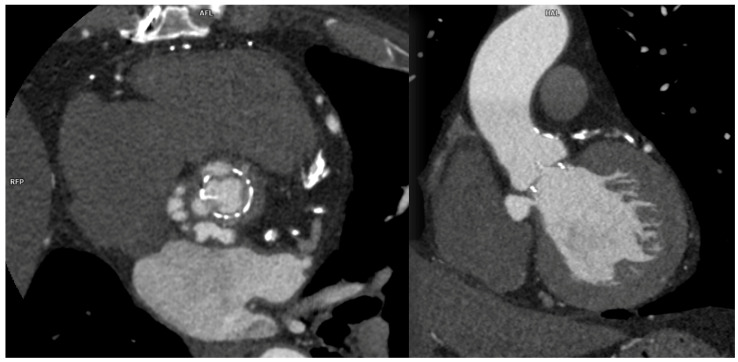
CCTA: A paravalvular leak can be noted in axial and coronal reconstructions in a patient after biological aortic valve placement.

In case of PMK implantation, right atrium or ventricular perforation with associated hemopericardium (Figure 5) was the most common complication (5/27 patients, 18.5%). All pacemakers included in the study had two or three intravenous leads. Cases of damage caused by PMK leads were diagnosed mostly in a chronic phase: 4 by 5 patients, more than 30 days after implementation. Only one case presented as an acute complication (<24 h after PMK placement), showing a more worrying and significant clinical picture, sustained by active bleeding in the pericardial sac.

The dislocation of the aortic prosthetic valve after a TAVI procedure was seen in two cases, with a symptomatic presentation manifestated by the patient as acute chest pain or thoracic discomfort.

**Figure 6:**
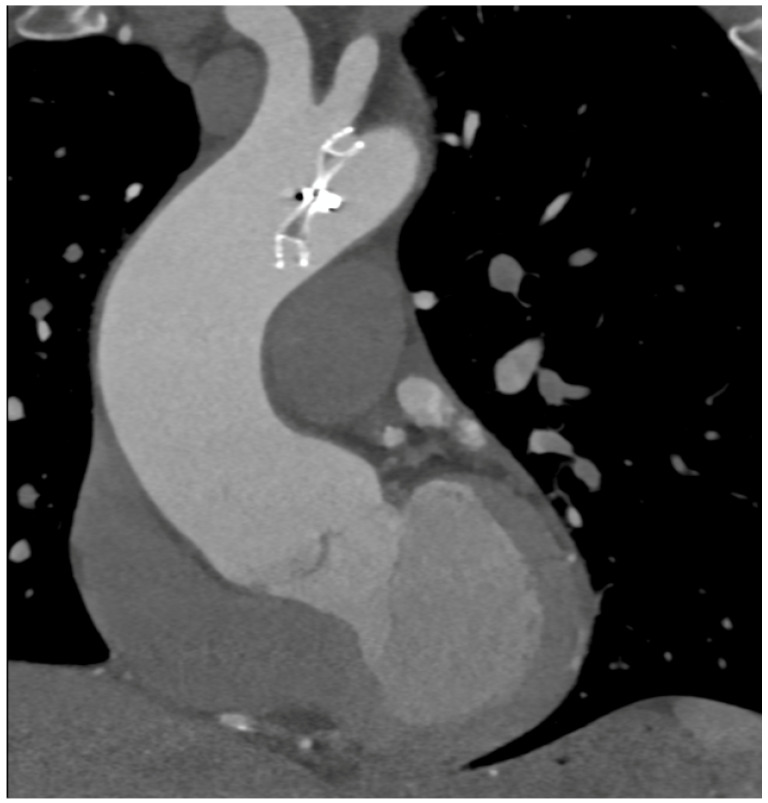
CCTA multiplanar reconstruction: Amplatzer septal occluder dislocated in the aortic arch.

In five patients who previously underwent aortic valve surgery, a non-emergency condition was diagnosed during a routine follow-up as an aortic root pseudoaneurysm (2/27 patients, 7.4%) or para-valvular leak (3/27 patients, 11.1%) (Figure 6).

**Figure 7:**
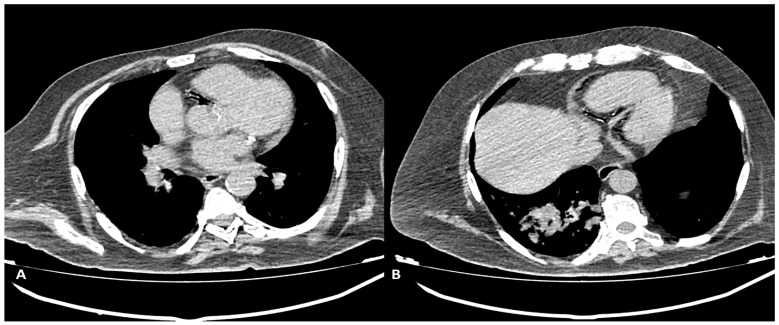
Non-contrast-gated CT, axial planes: air presence in the RCA and its branches can be noted. Scan was performed in the immediate follow-up after a CT-guided lung-biopsy procedure, in a symptomatic (chest pain and dyspnea) patient. (**A**) air bubbles in proximal RCA can be seen. (**B**) Air is present in the distal RCA and posterior descendent artery.

In one case (1/27 patients, 3.7%) of patent foramen ovale correction, we found an Amplatzer septal-occluder dislocation (Figure 7).

We encountered one case of intracoronary air embolism in a patient presenting with acute chest pain and dyspnea after a CT-guided lung biopsy procedure. Upon a CT-gated non-contrast scan, a significant number of air bubbles were found in the right coronary artery (mainly RCA) and its branches (Figure 7), due to transition through a pulmonary vein to the coronary circulation. CT acquisition was stopped after the non-contrast-gated preliminary acquisition, and the patient was immediately delivered to the cath lab for a coronary angiography.

## 4. Discussion

The primary endpoint of our study was to underline the importance of CCTA in the evaluation of cardiovascular post-procedural and post-surgical complications. Over time, thanks to the evolution of scanning and the implementation of post-processing software capabilities, CCTA has achieved a first-rate ranking in evaluating post-interventional patients presenting with a clinical suspect. On the other hand, it must be underlined that CT is still not considered in the guidelines flowchart, although it can quickly and accurately recognize severe or life-threatening complications in acute-care settings, allowing for immediate treatment. Moreover, it can assist in defining chronic post-operative complications (30 days to 6 months after the procedure) and guide clinicians for optimal management.

Previous studies examined the utility of CT in the evaluation of complications after endovascular procedures, demonstrating a wide range of percentages in terms of acute and chronic complications [17,18,19] and assessing how some of these occurrences may be benign while others are potentially fatal; on the other hand, this is the first report that explores the use of CT in almost all types of endovascular interventions on the heart, including the placement of cardiac pacing devices. This aspect amply justifies the high frequency of complications recorded in a chronic setting.

It must be taken into account that cardiac and noncardiac complications can occur at variable times after these procedures, with the clinical presentation ranging from asymptomatic to devastating. Invasive coronary angiography is the standard reference modality used in the evaluation of coronary arteries, with intravascular US and optical coherence tomography providing high-resolution information regarding the vessel wall.

In our study, all CCTA procedures were performed using ECG-gating with two different CT systems; we found some indubitable advantages in the dual-source scanner, which allowed for a faster CT acquisition and a reduction in motion and breathing artifacts. The most important aspect in cardiac CT imaging is the cardiac synchronization of the scan, which was adopted in the arterial phase in all our studies. This allows for a precise assessment, especially on cardiac structures and ascending aorta, decreasing nondiagnostic examinations caused by structural movement [2,17].

Even though we were using a faster scanner, we adopted drug premedication (β-blocker and nitrates) to all patients except those presenting specific contraindications.

In the 294 CCTAs examined, we found a total of 27 complications. Among them, coronary intimal dissection was found to be the most frequent (22.2%), in all cases associated with the ascending aorta.

Intimal dissection in the aortic or coronary wall during PCI is an infrequent complication characterized by the formation of a hematoma in the media and separation of the intimal layer, with compromise of the coronary blood flow. It can be explained either by direct trauma from the catheter tip or a vigorously expelled jet of contrast material from the catheter tip that abuts the wall of the coronary artery. Among risk factors that must be taken into account are female gender, mechanical trauma, technical factors such as the inappropriate positioning of the catheter tip in the coronary artery ostium, and overinflation of the angioplasty balloon. Ascending aortic dissection is more frequent during procedures involving RCA because of its smaller caliber and the hemodynamic force vector directed to the right convexity of the ascending aorta [20]. In our study, all cases of coronary artery dissection involved the RCA with the flap extension to the right valsalva sinus. CCTA can accurately define the aortic involvement guiding the correct management because limited aortic involvement may be treated by stenting the coronary dissection entry point, whereas extending aortic dissection distant from the coronary os may require surgical intervention [20]. The capability of CTA to precisely assess intimal flap extension and the presence or absence of intramural hematoma are fundamental to correct patient management. Moreover, a gated CTA scan is helpful for determining the coronary vessel patency impairment when present and consequently to establish the exact true and false lumen width.

Coronary pseudoaneurysms are relatively rare and can be caused by iatrogenic procedural trauma after PTCA or, more rarely, after coronary angiography. An aneurysm is defined as >1.5 times the normal arterial diameter, but pseudoaneurysms do not involve all layers of the vessel wall. Coronary pseudoaneurysms are mostly detected incidentally and are asymptomatic, but they can result in fistulae, rupture, bleeding, tamponade, and myocardial infarction; for these reasons, a prompt diagnosis is crucial [21], and CCTA allows for a correct definition of the size, morphology and location of the lesion.

We found coronary pseudoaneurysm in two patients (7.4%), both in the left coronary artery: one in the left main coronary artery (LMCA) and one in the left anterior descending (LAD), respectively.

Stent displacement, fracture or migration is a rare complication, and risk factors include the use of drug-eluting stents, manufacturing defects, or extensive vessel calcification. In the case of multiple overlapping stents, a possible inconvenience but hard to differentiate in CTA by struts fractures, is represented by stent misalignment or separation that can occur in the immediate post-procedural period or later. In both cases, the consequences are essentially the same and consist of the extravasation of the contrast material through the rupture, with a small collection of it adjacent to the stent.

We detected coronary stent misalignment or dislocation in a total of three patients (14%), which represents a high percentage, according to the literature [22]. All cases were diagnosed during routine follow-up scans to assess stent patency, in absence of patient-specific symptoms. Although in the literature, stent dislodgements are reported to be more common in the LMCA and LAD arteries, in our study, in all three cases, the RCA was involved. In one of the three cases, a patient underwent multiple stenting of the middle and distal RCA, and an unusual endoleak condition was diagnosed. A misalignment was found between two devices, with a consequential medium-contrast extravasation between stent meshes and the coronary vessel wall, configuring a type III endoleak.

Pacemaker implantation has become a widely used practice all over the world in a growing population of patients, but thanks to increased indications, complications may be related to pacemaker syndromes that represent the clinical manifestation of a suboptimal synchronization or a mechanical complication related to pacemaker positioning. Complications may be acute or subacute in the setting of pacemaker implantation; the more frequent among them are pneumothorax, cardiac perforation, pocket hematoma or bleeding, lead dislodgement, venous thrombosis, and mechanical lead complications. One of the most insidious inconveniences is represented by perforation of the myocardial wall, both in the acute and subacute phases. Symptoms of perforation include pleuritic chest pain from pericarditis, diaphragmatic or intercostal muscle stimulation, and, in the presence of pericardial effusion, patients may develop shortness of breath and hypotension as the tamponade develops [23].

Complications encountered after PMK implantation represented 18.5% of all cases in the study. One patient only (0.03%) was scanned in the acute phase, less than 24 h after PMK positioning, presenting with acute chest pain, dyspnea, and laboratory signs of anemization. CTA was fundamental in determining management because it clearly demonstrated acute bleeding and a significant hemopericardium. Four other patients presented at CT observation more than 30 days after leads implantation had been performed. They reported chronic complications and aspecific symptoms such as intermittent chest pain and dyspnea [24,25,26]. CT imaging confirmed the clinical or echocardiographic suspicion of cardiac wall perforation caused by the edge of leads. In chronic cases, the amount of heemopericardium was mild, and no signs of active bleeding were shown by CTA. PMK leads in these cases always determined a buffering effect on the wall laceration.

Complications of TAVI procedure can be immediate or periprocedural, and the latter can appear shortly after the procedure [13]. Prosthesis dislocation during TAVI is a rare but serious complication; the major complications following TAVI are considered to be mispositioning, valve migration/embolization, conversion to open surgery, need for pacemaker implantation, stroke, and myocardial infarction [27]. These complications can be managed effectively by implanting a second device and leaving the dislocated device safely in the aorta or by completely retrieving the valve [26].

Different scenarios can be encountered during the evaluation of a surgically implanted prosthetic valve. It must taken in account that the major problem can be represented by the biocompatibility of the prosthetic material, and CT is the optimal instrument to assess potential rejection issues [28,29].

ECG-gated CCTA plays an important role in the early diagnosis of local complications. CCTA examinations are carefully but also dynamically examined in all phases of the cardiac cycle to assess the valve leaflets and their movements, the intactness of the aortic anulus, and general complications such as hematomas. Stent dislocation after correct initial positioning is a very rare complication, but the valve can be dislocated to a too low position, determining significant hemodynamic paraaortic regurgitation, or too high, with overlap of the coronary ostia [13].

Surgical repair of atrial septal defects has essentially been replaced by percutaneous interventions. Even if these procedures are performed with excellent safety and efficacy in contemporary practice, some impairments have not completely been eliminated. Embolization of the device is still the most frequent complication, and its clinical presentation varies from an incidental finding to ventricular fibrillation and hemodynamic collapse. Amplatzer dislocation is a rare but serious event [30,31]. In the literature, device dislocation and embolism are ranges from 4% to 21%, and surgery is necessary in approximately 70% to 100% of those cases [30,31,32]. Clinically, a deficient aortic rim is considered a potential cause of device migration and further study; further studies in vivo and in silico can in the future aid in developing new prosthetic features to better fit aortic anulus morphology [32,33,34,35,36,37,38]. In one case (3.7%) of patent foramen ovale correction, we found an Amplatzer septal occluder (AGA Medical Corporation, Golden Valley, MN) dislocated in the aortic arch.

In one case we noted air in the RCA and its branches after a CT-guided lung biopsy procedure, without contrast-agent usage. This was the only case in which we did not perform CCTA but only gated non-contrast CT: the air in the coronary vessel and its branches stemmed from air transition in a pulmonary vein to the coronary circulation. The patient immediately underwent coronary angiography.

The use of the gated CTA in relation to the results of the present investigation should be recommended in the early post-procedural period, even if there is little suspicion of complications, given the high percentage of possible occurrences. If it is true that in some cases, complications have a limited impact on the management of the patient because they have a benign course, it is also true that only the CT scan has the possibility of accurately defining the origin and consequent instances of poor progression. In fact, often clinical evaluation alone or laboratory investigations and first-level imaging such as an ultrasound can only fuel suspicions that find a clear definition of their perimeter and their value upon the CT scan. This statement takes on greater value in consideration of the ever- lowering invasiveness of CT in terms of the reduced radiation dose that is administered and the smaller quantity of the contrast medium administered because of the use of modern scanners.

However, it must be recognized that for the large-scale use of a new diagnostic, the evidence of an improvement in diagnostic accuracy is not sufficient; rather, it is necessary to acquire the awareness of improvement in prognosis, something that the present examination does not demonstrate. It is therefore necessary to wait for randomized studies to be executed before being able with certainty to affirm the advisability of routinely introducing CT in the evaluation of patients in this specific setting.

## 5. Conclusions

Although the role of ECG-gated CCTA as an integral part of pre-procedural and surgical work-up is well known, there are still few studies in the literature and no recommendation for its use as an imaging technique in the follow-up of patients who have undergone coronary procedures and cardiac surgery.

The true incidence of cardiac post-procedural/surgical complications remains unrecognized and may often be underestimated. Our experience demonstrates that ECG-gated CCTA represents a fundamental tool to detect post-procedural endovascular/surgical complications in order to enable optimal patient management.

This investigation promotes CCTA cardiac synchronization as the imaging technique of choice for the diagnosis, management, and follow-up of post-procedural complications.

## Figures and Tables

**Table 1:** Demographic data and population characteristics according to CTA findings, categorized according to acute/chronic presentation and type of complication.

**Population**	**Males**	**Females**	**Age Range**					
	18	9	47–86 (68.3)					
	**Dissection**	**PMK Compl.**	**Valvular Int. Compl.**	**PSA**	**Stent Compl.**	**Air Embolism**	**Occluders Compl.**	**Total Compl**
Acute Pres.	6	1	2	-	-	1	-	
Chronic Pres.	-	4	5	3	4	-	1	
	6	5	7	3	4	1	1	27

Compl.: Complications; Int.: Interventions; PMK: Pacemaker; Pres.: Presentation; PSA: Pseudoaneurism.

## Data Availability

Available on request.

## Abbreviations

CAD	Coronary artery disease
CCTA	Coronary Computed Tomography Angiography
TAVI	Transcatheter aortic valve implantation
i.v.	intra-venous
PMK	Pacemaker
PCI	Percutaneous coronary intervention
RCA	Right coronary artery
LMCA	Left main coronary artery
LAD	Left anterior descending
